# Electrical Remodeling in Right Ventricular Failure Due to Pulmonary Hypertension: Unraveling Novel Therapeutic Targets

**DOI:** 10.3390/ijms24054633

**Published:** 2023-02-27

**Authors:** John F. Park, Justine Liang, Soban Umar

**Affiliations:** Department of Anesthesiology and Perioperative Medicine, University of California, Los Angeles, CA 90095, USA

**Keywords:** right ventricle, pulmonary hypertension, arrhythmia, ion channel, RNA sequencing, transcriptome, drug repurposing, Sugen-hypoxia, monocrotaline

## Abstract

Arrhythmias in the setting of right-ventricular (RV) remodeling contribute to majority of deaths in patients with pulmonary hypertension. However, the underlying mechanism of electrical remodeling remains elusive, especially ventricular arrhythmias. Here, we analyzed the RV transcriptome of pulmonary arterial hypertension (PAH) patients with compensated RV or decompensated RV and identified 8 and 45 differentially expressed genes known to be involved in regulating the electrophysiological properties of excitation and contraction of cardiac myocytes, respectively. Transcripts encoding voltage-gated Ca^2+^ and Na^+^ channels were notably decreased in PAH patients with decompensated RV, along with significant dysregulation of K_V_ and K_ir_ channels. We further showed similarity of the RV channelome signature with two well-known animal models of PAH, monocrotaline (MCT)- and Sugen-hypoxia (SuHx)-treated rats. We identified 15 common transcripts among MCT, SuHx, and PAH patients with decompensated RV failure. In addition, data-driven drug repurposing using the channelome signature of PAH patients with decompensated RV failure predicted drug candidates that may reverse the altered gene expression. Comparative analysis provided further insight into clinical relevance and potential preclinical therapeutic studies targeting mechanisms involved in arrhythmogenesis.

## 1. Introduction

Pulmonary arterial hypertension (PAH) develops as progressive remodeling of pulmonary arteries that leads to elevated pressure in the pulmonary circulation and right ventricular (RV) remodeling. The mechanism of RV remodeling is a complex interplay of cardiomyocyte hypertrophy, fibrosis, metabolic changes, angiogenesis, electric remodeling, and inflammation [1,2]. The overall RV function and arrhythmias are important contributors to the overall morbidity and mortality in PAH patients. The prevalence of arrhythmias is predicted to be 15–20%, and sudden cardiac death accounts for majority of deaths (28%) aside from acute RV failure [3,4]. More specifically, ventricular arrhythmias are considered one of the major causes of sudden cardiac deaths in pulmonary hypertension patients with an estimated prevalence of 8% to 26% [3,4].

Clinically, arrhythmias are associated with worsening cardiac function in PAH, and the presence of a prolonged QT is associated with increased frequency of ventricular ectopics [4,5]. Despite advancements in medical therapy for PAH, there is still a paucity of effective antiarrhythmic therapies. Current pharmacological management of ventricular arrhythmias in PAH are limited due to potential side-effects and/or risk of hemodynamic deterioration. Thus, it is imperative to further understand the molecular mechanisms involved in PAH-induced ventricular arrhythmias.

Prior preclinical studies in rats have shown evidence of electrical remodeling in the setting of RV failure such as conduction velocity slowing, increased RV action potential duration, and QT prolongation [6,7]. Monocrotaline toxin (MCT) and VEGF (vascular endothelial growth factor) receptor antagonist (SU5416) combined with chronic hypoxia followed by normoxia (SuHx) are two well-known animal models that induce PAH and RV failure in response to pressure overload [8]. Recent transcriptomic analysis of RV from MCT and SuHx rats revealed dysregulation of biological pathways involving cardiac conduction [9]. Furthermore, the RV transcriptome and proteome of PAH patients with decompensated RV failure revealed similar findings [10]. We aim to highlight the translational similarities across species as well as utilize known genes involved in electrical remodeling isolated from two rat models of PAH and human RV tissue. We hope to identify potential drug therapies that target RV arrhythmias.

## 2. Results

We analyzed the transcriptome of PAH patients with compensated (RV-C, *n* = 11) or decompensated RV (RV-D, *n* = 11) compared to healthy donor RV (RV-CTRL; *n* = 17) from a public transcriptomic database (GEO Accession Number GSE198618). The clinical information of human RV tissue for transcriptomic analysis is described in [10]. Gene set enrichment analysis of patients with decompensated RV compared to RV-CTRL showed a significant downregulation of biological processes involved in regulating cardiac conduction and ventricular cardiac muscle cell action potential in both the transcriptomic and proteomic datasets [10]. Given these findings, we sought out to further analyze the transcriptome signature of ion channels that are known to be involved in regulating the electrophysiological properties of excitation and contraction of cardiac myocytes.

**Figure 1:**
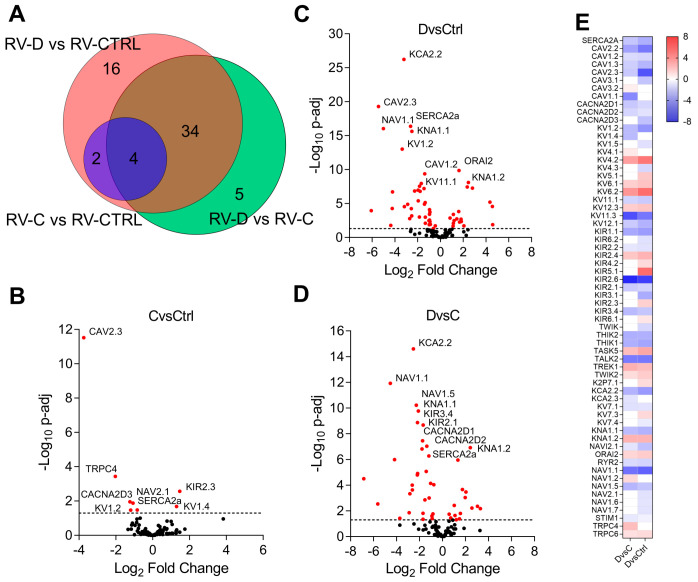
Channel gene expression profile analysis of PAH patients with compensated (RV-C) or decompensated RV (RV-D) compared to healthy donor RV (RV-CTRL). (**A**) Venn diagram of overlapping DEGs from RV-D, RV-C, and RV-CTRL patients. (**B**–**D**) Volcano plot of DEGs from RV-C versus RV-CTRL, RV-D versus RV-CTRL, and RV-D versus RV-C. The horizontal dash line represents False Discovery Rate-corrected *p*-value cutoff <0.05. Red points represent statistically significant DEGs. (**E**) Heat map of statistically significant DEGs from the following groups: RV-D versus RV-CTRL; RV-D versus RV-C. Blue or red represents downregulation or upregulation, respectively.

We curated a list of ion channels (“channelome”) that consist of the following families: voltage-gated K^+^ channels (K_V_), inward-rectifier K^+^ (K_ir_), two-pore forming K^+^ channels (K_2P_), Ca^2+^ activated (K_Ca_), voltage-gated Na^+^ channels (Na_V_), voltage-gated Ca^2+^ channels (Ca_V_), transient receptor potential canonical (TRPC), Ca^2+^ release-activated Ca^2+^ channel protein (ORAI), ryanodine receptor (RyR2), and sarco/endoplasmic reticulum Ca^2+^-adenosine triphosphatase (SERCA). We filtered the relative differentially expressed genes (DEG) in PAH patients with compensated or decompensated RV (Supplementary Tables S1–S3). Among the 115 channel genes, 56 genes were found differentially expressed between RV-D versus RV-CTRL with a *p*-adjusted value < 0.05. In addition, 45 and 8 DEGs were identified between RV-D versus RV-C and RV-C versus RV-CTRL, respectively (Figure 1). The majority of DEGs encoding voltage-gated Ca^2+^ and Na^+^ channels were notably decreased in patients with decompensated RV (Figure 1). In contrast, there was significant dysregulation of K_V_ and K_ir_ channels. The expression of DEGs when comparing RV-D patients with either RV-CTRL or RV-C showed significant overlap by 38 DEGs.

**Figure 2:**
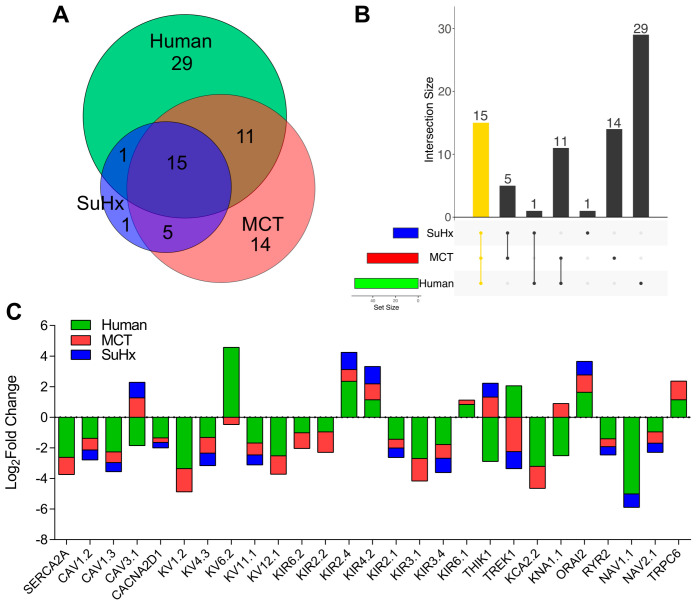
Channel gene expression profile analysis of MCT, SuHx, and PAH patient with decompensated RV. (**A**) Venn diagram of overlapping dysregulated DEGs from MCT, SuHx, and Human (RV-D versus RV-CTRL). (**B**) UpSet plot of the intersection between MCT, SuHx, and Human. Yellow represents overlap of SuHx, MCT, and Human. (**C**) Stacked graph showing Log2 fold change of statistically significant DEGs present in MCT (red) and/or SuHx (blue) with human (green).

Next, we performed comparative transcriptomic analysis of RV dysfunction between RV-D and two animal models of PAH-induced RV failure. We analyzed the DEGs from MCT and SuHx rat datasets as previously described in [9] (Supplementary Tables S4 and S5). Both MCT and SuHx rats had significantly elevated right ventricular systolic pressure (MCT: 90.6 ± 16 mmHg; SuHx: 90.8 ± 18.7 mmHg), decreased RV fractional area change (MCT: 18.6 ± 10.8%; SuHx: 15.8 ± 5.7%), and increased RV hypertrophy index (MCT: 0.7 ± 0.2; SuHx: 0.6 ± 0.2) [9]. Among the 56 DEGs from RV-D versus RV-CTRL, 16 out of 22 DEGs from SuHx overlapped (Figure 2). In contrast, 26 out of 45 DEGs from MCT overlapped with RV-D. We identified 15 DEGs common among MCT, SuHx, and human. Of note, Ca_V_3.1, K_V_6.2, THIK1, TREK1, and KNa1.1 from MCT and SuHx were discordant with decompensated RV patients (Figure 2; Supplementary Table S6).

**Figure 3:**
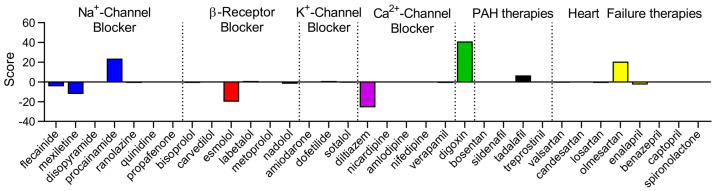
Screening of channelome against pharmacological perturbagens tested in human cell lines (CMap). Graph showing connectivity scores of DEGs from RV-D versus RV-CTRL to common anti-arrhythmic drugs (Na^+^—channel blockers, β—receptor blockers, K^+^—channel blockers, and Ca^2+^—channel blockers), and PAH and heart failure therapies. Negative or positive score predicts anti- or pro-arrhythmic potential, respectively. Connectivity score scale −100 to 100.

To identify anti-arrhythmic potential of existing drugs for PAH patients with decompensated RV, we screened the channelome signature against a large reference of gene differential expression database generated by perturbing cell lines (Connectivity Map; CMap). We applied cutoff of log2 fold change > 1.5 and an adjusted *p*-value < 0.05 for the analysis. As predicted, the commonly used anti-arrhythmic drugs within class I (Na^+^—channel blocker), class II (β-receptor blocker), class III (K^+^-channel blocker), and class IV (Ca^2+^-channel blocker) showed poor connectivity scores except for diltiazem and esmolol (Figure 3). Digoxin had a positive connectivity score and thus suggests pro-arrhythmic potential. Other medications used for PAH and heart failure also showed poor connectivity scores (Figure 3). We provided a list of potential existing drugs and drug-targets with strong anti-arrhythmic potential such as Bruton’s tyrosine kinase (BTK) inhibitor, transient receptor potential polysystin sub family (TRPP) antagonism, and β3-adrenergic receptor agonism (Supplementary Table S7).

## 3. Discussion

In this study, we examined the dysregulation of ion channel expression in patients with decompensated RV and compensated RV in the setting of PAH that may play a role in ventricular arrhythmogenesis. Our results showed downregulation of genes encoding voltage-gated Ca^2+^ and Na^+^ channels and dysregulation of various K^+^ channel sub-types that likely contribute to ventricular arrhythmias. Additionally, we compared the RV human channelome with MCT and SuHx rats to determine human relevance. We identified similarities and unique differences with RV-D patients. Finally, we used the channelome from RV-D patients to identify potential drug-repurposing candidates.

Clinically, prolongation of QT interval and action potential duration (APD) has been associated with maladaptive RV patients in the setting of PAH [3,5]. Downregulation of repolarizing voltage-gated K^+^ channels such as K_V_1.2, K_V_1.5, and K_V_4.3 in the RV can lead to increased APD or susceptibility to arrhythmias, which is consistent with preclinical studies in RV hypertrophied rats [11,12]. Other studies have shown the downregulation of K_V_ channels in the setting of hypoxia-mediated oxidative stress [13]. Similarly, K_ir_ family has been extensively studied in the setting of sudden cardiac deaths, associated genetic syndromes such as prolonged QT syndrome, and heart failure. For instance, K_ir_1.1, K_ir_2.1, and K_ir_3.1 channels are responsible for maintaining K^+^ homeostasis and establishing the resting membrane potential. However, the downregulation of K_ir_ channels leads to membrane potential or trigger activity instability, delayed after depolarizations, and/or cardiomyocyte dysfunction [12,14].

Ca^2+^ ions play a significant role in regulating cardiac conduction and mechanical contraction. Alterations of Ca^2+^ influx or Ca^2+^ homeostasis by ion channels and/or Ca^2+^-dependent signaling pathways can lead to electrical remodeling. Ca_V_1.2 is the most abundant Ca^2+^ channel in cardiac myocytes, and it couples excitation to contraction [15]. *CACNA2D1* gene encodes for the α_2_δ_1_-subunit of Ca_V_1 and Ca_V_2 channels, which regulates Ca^2+^ current density and ventricular cardiac contraction. Loss of function mutations of Ca_V_1.2 or *CACNA2D1*, such as Brugada syndrome, can lead to a reduction in Ca^2+^ currents and short QT intervals, which increases the risk for arrhythmias and sudden cardiac death [16]. Ca_V_3.1 has been shown to be exclusively expressed within the cardiac conduction system and thereby regulates cardiac pacemaker activity and impulse conduction. A recent study showed that the loss of Ca_V_3.1 promotes bradycardia-related ventricular arrhythmias following atrioventricular blockade [17].

Cardiac voltage-gated Na^+^ channels play a critical role in the fast depolarization phase of action potential and excitation-contraction coupling. Na_V_1.5 is the predominant isoform of voltage-gated Na^+^ channels in cardiac myocytes, and it is downregulated in PAH patients with decompensated RV failure [18]. Dysregulation of Na_V_1.5 has been reported in various cardiac channelopathies such as prolonged QT syndrome, Brugada syndrome, idiopathic ventricular fibrillation, and arrhythmogenic right ventricular cardiomyopathy (ARVC) [18,19]. Based on studies in ARVC, disrupting the interaction of Na_V_1.5 with desmosomes or connexins can result in decreased Na^+^ current density and conduction velocity. This leads to an impairment in mechanical and electrical coupling, which may partially explain the increased risk for fatal arrhythmias in patients with ARVC [19]. Interestingly, desmoglein-2 and connexin-43 (Na_V_1.5 interacting proteins) are downregulated in RV-D transcriptome [10]. Further studies are needed to identify the role of Na_V_1.5 and its interacting proteins in ventricular arrhythmias.

Our drug repurposing analysis based on CMap using the channelome predicted poor therapeutic potential of common anti-arrhythmic drugs in treating PAH-related arrhythmias. Diltiazem (Ca^2+^ channel blocker) and Esmolol (β-blocker) showed possible therapeutic potential in reversal of arrhythmias, but it is likely limited by its negative inotropic and chronotropic effects [20]. PAH patients with RV dysfunction have a fixed stroke volume and are highly dependent on heart rate to increase cardiac output. Interestingly, three small prospective clinical trials and animal models have shown potential safety of β-blocker use in pulmonary hypertension [21]. In addition, Ca^2+^ channel blockers are currently trialed in patients with PAH due to their positive vasoactive response [22].

While the current mainstay anti-arhythmic drugs were not predicted to be effective, our drug repurposing analysis revealed other classes of drugs that have potential in reversing the channelome signature of RV decompensated PAH patients. Bruton’s tyrosine kinase (BTK) inhibitor was one of the top perturbagens identified in our analysis. BTK is a non-receptor protein-tyrosine kinase that has been implicated in proliferation of malignant B-cells. Ibrutinib, an FDA-approved first-generation irreversible BTK inhibitor for B-cell malignancies, has been associated with various cardiovascular adverse events such as atrial arrhythmias secondary to its off-target effects [23,24]. The purposed mechanism of ibrutinib-induced atrial arrhythmias involves dysregulation of phosphoinositide 3-kinases (PI3K) activity, which is responsible for regulating various ion channels such as Ca_V_1, K_ir_, and Na_V_1.5 [24,25]. For instance, inhibition of PI3K activity causes activation of late Na^+^ and L-type Ca^2+^ current that eventually leads to prolongation of action potentials and early/delayed afterdepolarizations [25]. Gene set enrichment analysis of RV-D patients compared to RV-CTRL showed a significant upregulation of PI3K signaling pathway, which was not seen in MCT or SuHx rats [9,10]. Interestingly, a recent study showed an upregulation of PI3K signaling in the lungs of both PAH patients and rats. Inhibition of PI3K catalytic subunit p110α reversed pulmonary vascular remodeling, RV systolic pressure, and RV hypertrophy in SuHx and MCT rats [26]. To the best of our knowledge, there are limited studies on the role of PI3K signaling in RV arrhythmogenesis in the setting of PAH. While BTK inhibition has been associated with atrial arrhythmias, there are conflicting clinical data showing QT prolongation or increased risk for ventricular arrhythmias with BTK inhibitors [24]. Further studies elucidating the electrophysiological mechanism of BTK inhibition in the setting of RV dysfunction and ventricular arrhythmias are needed.

Other top predicted drug classes include transient receptor potential polycystin sub family (TRPP) antagonism and β3-adrenergic receptor agonism. TRPP channels are nonselective cation channels that regulate Ca^2+^ homeostasis and excitation-contraction coupling by their interaction with RyR2 [27]. They are expressed in endothelial cells, vascular smooth muscle cells, and myocytes [28]. Recent studies in patients with autosomal dominant polycystic kidney disease (ADPKD) showed that ablation of cardiac TRPP channels leads to an upregulation of action potential-repolarizing K^+^ currents, decrease in L-type Ca^2+^ channel density, and reduction in APD and SERCA activity [29,30]. These findings may in part explain the increased risk of arrhythmias seen in ADPKD patients. Similarly, β3-adrenergic receptor agonism has been shown to suppress ventricular tachyarrhythmias [31] by a mechanism that involves regulation of Ca^2+^ homeostasis by nitric oxide signaling-dependent inhibition of L-type Ca^2+^ current and/or increase in Na^+^/K^+^ pump activity [32]. Collectively, both predicted drug targets highlight the importance of normalizing intracellular Ca^2+^ by modulation of associated proteins involved in sarcoplasmic reticulum Ca^2+^ release. Further investigations in managing Ca^2+^ homeostasis for ventricular arrhythmias in PAH patients are warranted.

Significant remodeling of RV in the setting of PAH such as extracellular matrix proteins, ion channels, and intracellular Ca^2+^ handling is well established in both human and animal models [12,33]. For instance, the dysregulation of K_V_6.2, ORAI family, and TRP genes has been associated with cardiac fibrosis-related arrhythmias [34]. Dysregulation of K_V_6.2 and ORAI in cardiac fibroblast can directly affect electrically coupled myocytes and alter cardiac action potentials [34]. While dysregulation of ion channels directly contributes to the development of arrhythmias, alterations to cardiac fibrosis likely behave as a pro-arrhythmic substrate. Clinically, patients with systemic RV fibrosis as detected by magnetic resonance imaging showed an association with adverse arrhythmias and sudden cardiac deaths [33,35]. The increase in collagen fibers between cardiomyocytes likely influences the channelome responsible for the electrical uncoupling of cardiomyocytes leading to cardiac conduction disturbances. Further studies are needed to provide evidence of direct contribution of RV fibrosis as an arrhythmogenic substrate.

We identified Ca_v_3.1, K_v_6.2, THIK1, TREK1, and KNa1.1 in MCT and SuHx rats to be discordant with decompensated RV patients. These are known genes involved in pressure-overload induced cardiac hypertrophy, end-stage heart failure, supraventricular tachycardias associated with heart failure, or cardiac hypertrophy-mediated electrical changes [7,12]. These discordant genes may indicate divergent functions or marked differences in cardiac physiology between rodents and decompensated RV patients. MCT and SuHx animal models reflect decompensated RV in its purest form and do not reflect other contributing factors seen in PAH patients with RV dysfunction such as other disease processes or comorbidities. Concomitant medication uses in treating PAH and heart failure—such as diuretics, Ca^2+^ channel blockers, prostacyclin analogs, endothelin receptor antagonist, and/or phosphodiesterase inhibitors—are not influencing gene expression in the animal models. This is an inherent limitation of analyzing transcriptomic signatures from patients with pathological diseases. Furthermore, we cannot exclude the possibility that altered gene expression will directly translate to proteins. Further validation and function assays will be needed to confirm our findings.

Furthermore, the differences in gene expression between animal models and humans may be due to the known side-effects of the toxins used to induce PAH and RV failure. For instance, MCT is known to have off-target effects such as myocarditis that could limit the use of MCT model for studying arrhythmias. It is not surprising that about ~31% of DEGs (14 out of 45) are unique to MCT as opposed to SuHx (4%; 1 out of 22 DEGs) from our analysis (Figure 2). These findings suggest SuHx as an adequate PAH model for studying arrhythmias as it could avoid the limitations of MCT. Alternatively, a pulmonary artery banding (PAB) model could be used as well since it avoids the systemic or toxic effects of MCT and SuHx. However, it is difficult to induce severe RV failure in a PAB animal model, and it lacks severe pulmonary vascular remodeling [8].

In conclusion, the RV remodeling seen in PAH patients due to heart failure, fibrosis, oxidative stress, or direct alteration of the ion channel milieu may be the substrate for malignant ventricular arrhythmias. We organized a list of potential ion channels that are likely involved in arrhythmogenesis. We identified a significant dysregulation of Na_V_, Ca_V_, K_V_, K_ir_, K_2P_, K_Ca_, TRP, and ORAI channels in PAH patients with decompensated RV, which was compared to those in preclinical PAH models, MCT and SuHx. We hope to further facilitate investigation of arrhythmias using the appropriate preclinical PAH models and to identify new anti-arrhythmic drugs for PAH patients.

## 4. Materials and Methods

Differential expression data were downloaded from the publicly available Gene Expression Omnibus (GEO) dataset GSE198618. The dataset contains RNA-sequencing results of RV tissues obtained from patients with normal RV function (*n* = 17), compensated RV function (*n* = 11), and decompensated RV function with end-staged PAH (*n* = 11). Detailed clinical information is described in [10]. Differential expression analysis was performed using the DESeq2 R package and R script provided by [10]. Differentially expressed genes with False Discovery Rate < 0.05 were considered statistically significant.

Differential expression data of MCT (*n* = 4), SuHx (*n* = 4), and healthy control rats were obtained from prior RNA-sequencing dataset provided in [9]. Detailed explanation of protocol and procedures in obtaining RV tissue from PAH animal models are described in [9]. In brief, adult male *Sprague–Dawley* rats received either a single subcutaneous injection of endothelial toxin monocrotaline (MCT; 60 mg/kg), vascular endothelial growth factor receptor antagonist Sugen (SU5416; 20mg/kg) with three-weeks of hypoxia (10% oxygen) followed by 2 weeks of normoxia, or phosphate buffer saline for control. Transthoracic echocardiography was performed to monitor cardiopulmonary hemodynamics using a Vevo 2100 high-resolution image system (FUJIFILM VisualSonics, Toronto, ON, Canada). The right ventricular systolic pressure was measured directly by a catheter connected to a pressure transducer (ADInstruments, Oxford, United Kingdom) into the RV four weeks after MCT and five weeks after Sugen injection. MCT and SuHx RV tissue was collected for transcriptomic analysis after confirming severe RV dysfunction with echocardiography and direct RV catheterization.

Transcriptomic signature of patients with decompensated RV were queried against the full connectivity map dataset (CMap; Broad Institute, Cambridge, MA, USA), which contains differential expression patterns from perturbing human cell lines [36]. Perturbagens with positive or negative connectivity scores have similar or opposite signatures to that of the query.

## Data Availability

The transcriptomic analysis of compensated and decompensated human right ventricles from pulmonary arterial hypertension patients can be found using the publicly available Gene Expression Omnibus (GEO) dataset GSE198618 (https://www.ncbi.nlm.nih.gov/geo/query/acc.cgi?acc=GSE198618). The data was access on 25 September 2022. The transcriptomic analysis of decompensated right ventricles from monocrotaline and Sugen-hypoxia rats can be found in the Supplementary Materials from the following publication [9].

## Supplementary Materials

The following supporting information can be downloaded at: https://www.mdpi.com/article/10.3390/ijms24054633/s1.
